# Numerical simulation of micro crack evolution and failure modes of limestone under uniaxial multi-level cyclic loading

**DOI:** 10.1038/s41598-023-31360-x

**Published:** 2023-03-13

**Authors:** Yanjun Yin, Jianhua Hu, Guanping Wen, Xiao Xu, Pingping Zeng

**Affiliations:** 1 School of Resources and Safety Engineering, Central South University, Changsha, 410006 China; 2 Zijin School of Geology and Mining, Fuzhou University, Fuzhou, 350108 China

**Keywords:** Civil engineering, Mechanical engineering

## Abstract

Deep rock structures are often subjected to complex cyclic disturbances generated by earthquakes and blasting vibrations. The rocks will resist disturbance with multiple stress levels, and the research on mechanical response is still insufficient under such conditions. A series of multi-level cyclic loading experiments were subjected to limestone specimens to obtain the stress–strain relation and fracture behavior. This study explored the effect of amplitude and cycle times on rocks. A Discrete Element Method model of rock specimens was established in Particle Flow Code 2D (PFC^2D^). The simulation results are coincidental with the experiment results. The results show that loading with low cycles can strengthen the rock, but loading with high cycles will present deteriorated effect on the rock. In the numerical simulation test, the initial crack will appear earlier with the amplitude increase. More micro cracks will be induced as the number of cycles per level increases. Moreover, tensile cracks are mainly distributed around the specimen when shear cracks widely appear in the central area. With the increase of amplitude, failure modes with mixed shear and tensile cracks will become universal.

## Introduction

Various deep rock engineering, such as rock pillars, tunnels, and caverns, are often subjected to dynamic disturbance generated by earthquakes, blasting vibrations, and other repetitive loadings^1,2^. The stability of the rock structure is usually weakened under dynamic loading and causes fatigue damage^3–6^. Hence, the investigation of damage process and fracture behavior under dynamic loading is significant to ensure the stability of rock engineering structures.

Cyclic loading is commonly used in the laboratory to simplify the action of complex stress disturbance on rocks^7–9^. Some typical characteristic factors of cyclic loading, including amplitude, confining pressure, loading rates, strain rates, loading frequency, etc., have been extensively studied for their damage mechanisms on rocks^10–15^. Most research focuses on the condition that the amplitude and stress level is constant during the loading process. However, considering the process of stress growth in the disturbance process, such as multi-level stress waves acting on the rock mass during earthquakes. Therefore, it is necessary to adopt a multi-level cyclic loading experiment. Its characteristic is that the stress level will rise with the increase in loading level. At present, few types of research have been carried out in this field. Peng et al.^16,17^ studied the failure mechanism of sandstones under multi-level cyclic loading from the crystal rotation perspective and the rock mass distribution after failure. Zhang et al.^18^ revealed that inhomogeneous damage would concentrate on the top and bottom of the coal and gradually propagate to the middle part during the multi-level cyclic loading. Some researchers also studied the fracture behavior of rock under graded cyclic loading and obtained significant results^19–23^. The above studies help grasp the failure mechanism of the rocks.

Numerical simulations provide an effective and convenient method for studying the failure processes of the rock mass. Various numerical simulation methods, including FLAC^3D^^24^, PFC^25^, RFPA^26^, 3DEC^27^, COMSOL^28^, etc., have been extensively applied in simulating the failure characteristics of rock under cyclic disturbance conditions. Based on the Discrete Element Method (DEM), PFC primarily simulates microscopic fracture processes and crack evolution in rock specimens, which has been verified by many studies^29–32^. Xu et al.^33^ used PFC^3D^ to investigate the cracks distribution characteristics and the damage evolution process of rock joints by the cyclic shear loading tests. Zhou et al.^34^ conducted a simulation in PFC^2D^ to research the crack propagation mechanisms of granite under ultrahigh-frequency cyclic loading. Moreover, to improve the accuracy of simulating the damage characteristics of rock materials under complex stress conditions, some researchers innovated some contact models and implemented them in PFC^35–37^. Song et al.^38^ presented a stress corrosion model, which can simulate geomaterials’ mechanical behavior under multi-level cyclic loading in PFC^3D^. Shi et al.^39^ put forward a damage model for sandstone under complex coupled cyclic loading. They studied the damage evolution by monitoring the micro force field provided by PFC^2D^.

For the relevant mechanical responses of rocks under multi-level loads, most previous studies focused on the effect of loading factors on various mechanical parameters, such as fatigue strength, dynamic elastic modulus, and Poisson’s ratio. It should be noted that the relevant research about the crack evolution and failure mode of rock under multi-level cyclic loading is still insufficient, especially from the perspective of numerical simulation.

This study subjected a series of laboratory multi-level cyclic loading experiments with different amplitude and loading cycles to limestone specimens. A PFC^2D^ simulation was conducted to reproduce the mechanical properties according to the experiment results. The displacement field evolution and crack evolution were further investigated from the microscopic perspective. In addition, the failure mode of specimens was also discussed based on the simulated results.

## Materials and methods

### Materials

**Figure 1:**
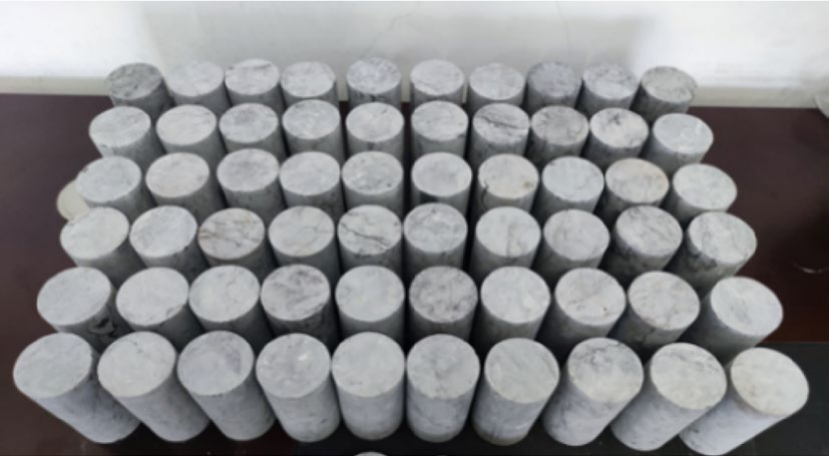
Limestone specimens.

**Figure 2:**
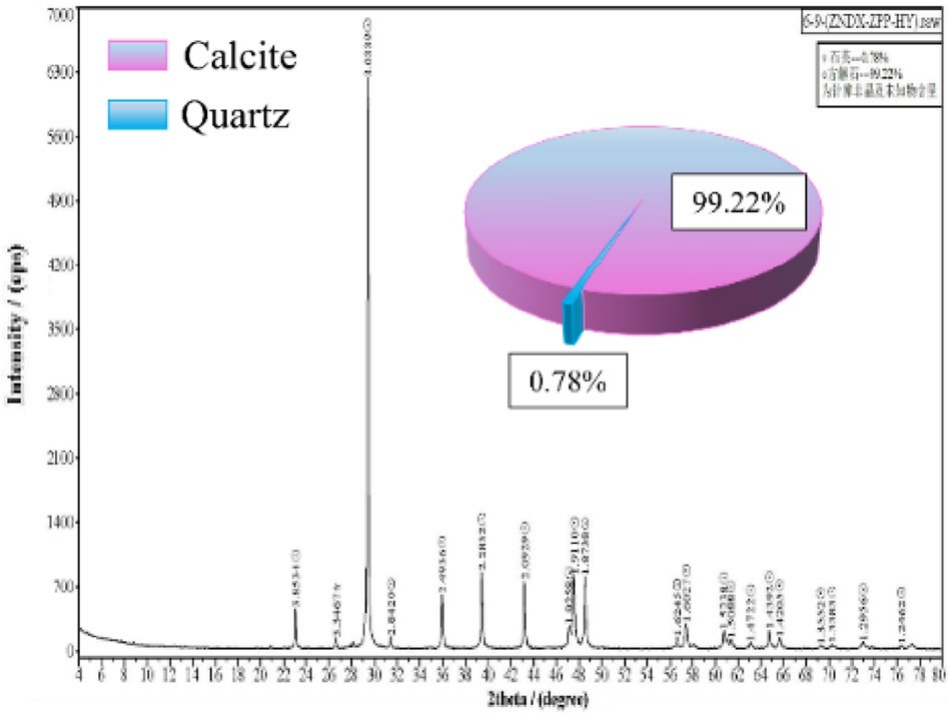
The composition results based on XRD.

As shown in Fig. 1, cylindrical limestone specimens with dimensions of 50 × 100 mm were obtained from a deep mine, where the pillars at the depth were exposed to stress disturbance. No apparent fractures or defects were observed on the surface of the specimens. The XRD results show that the main compositions of the specimens are calcite (99.22%) and quartz (0.78%), as shown in Fig. 2. Some principal physical and mechanical parameters of the specimens are listed in Table 1.

**Table 1:** Principal physical and mechanical parameters of limestone.

Density/kg m^−3^	*E*/GPa	Poisson’s ratio	P wave velocity/m s^−1^	Porosity/%	UCS/MPa
2710	87.88	0.21	2850	0.48	117.25

### Experimental methods

**Figure 3:**
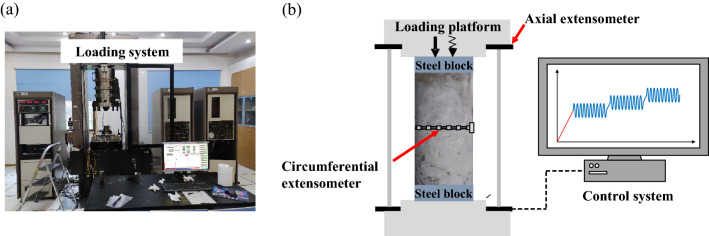
Experiment scheme: (**a**) Mechanics testing system-815; (**b**) Testing diagram.

A Mechanics Testing System-815 (MTS-815) was used for uniaxial multi-level compression experiments, as shown in Fig. 3a. Besides, an axial extensometer and a circumferential extensometer in contact with the specimen were equipped, as shown in Fig. 3b. Sine wave was selected as disturbance waveform, which could well model the disturbing effects induced by blasting and seismic^40,41^.

**Figure 4:**
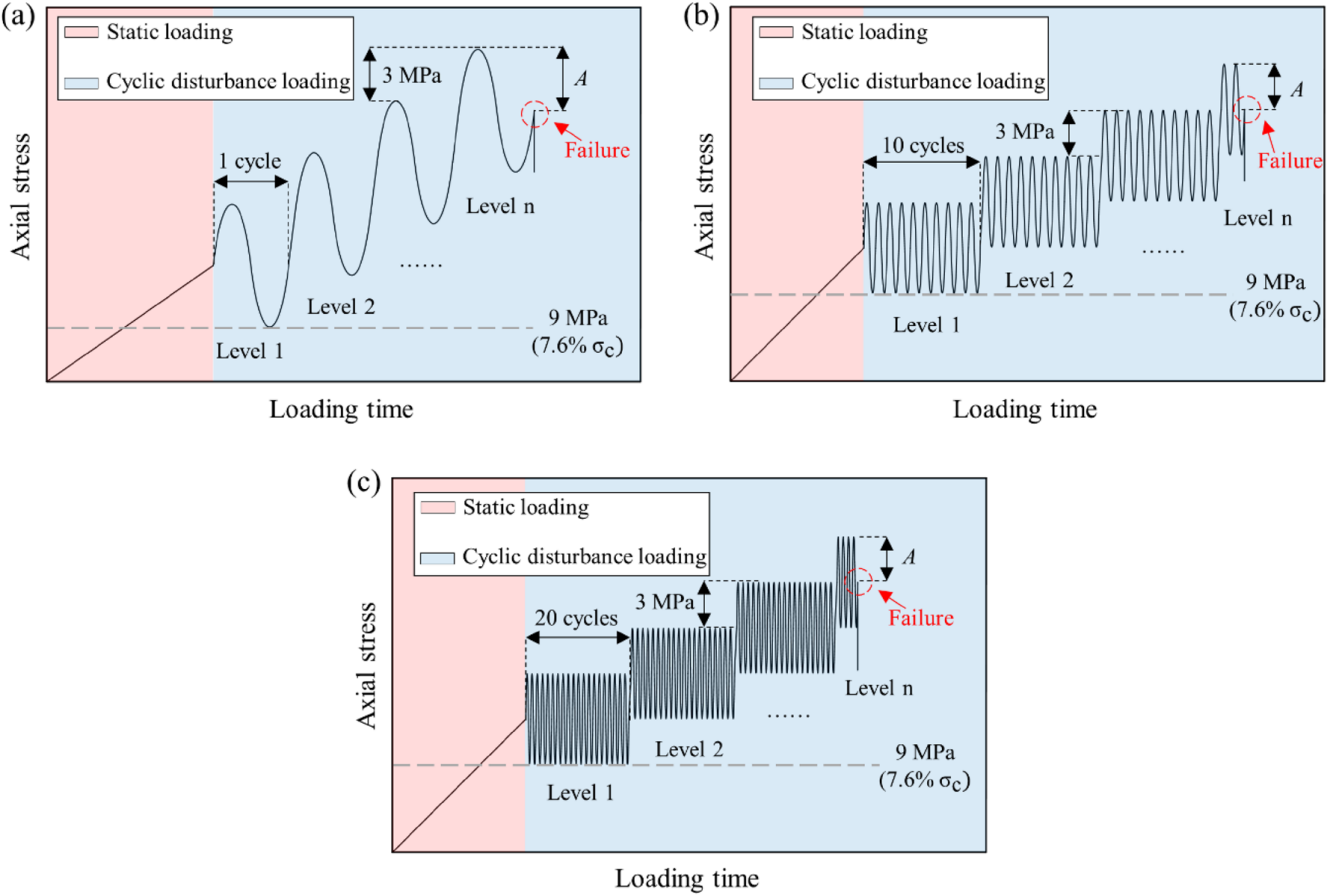
Schematic diagram of loading paths (**a**) Path in *N* = 1; (**b**) Path in *N* = 10; (**c**) Path in *N* = 20.

The limestone specimens were divided into nine test groups. Each group of tests shall be conducted three times to avoid errors. The loading paths consist of two parts: static loading and cyclic disturbance loading, as shown in Fig. 4. In the static loading phase, the axial stress was applied to the specimens at a rate of 0.5 kN/s until the stress reached the initial stress (${\text{I}}_{{\text{S}}} )$. Subsequently, cyclic stress was applied to the limestone specimens. To make the rock compact in the static loading stage, amplitude (*A*) and loading cycles corresponding to each stress level (*N*) are listed in Table 2. The frequency of the sine waveform was set to 0.1 Hz. Between every two subsequent loading levels, the axial stress was increased by 3 MPa at a rate of 0.5 kN/s.

**Table 2:** The testing schemes for multi-level cyclic loading.

Groups	Initial stress (${\text{I}}_{{\text{S}}} )$/MPa	Amplitude (*A*)/MPa	Loading cycles in each level (*N*)
F-4.5-1	13.5 (11.5% ${\upsigma }_{{\text{c}}}$)	4.5 (3.8% ${\upsigma }_{{\text{c}}}$)	1
F-9.0-1	18.0 (15.3% ${\upsigma }_{{\text{c}}}$)	9.0 (7.6% ${\upsigma }_{{\text{c}}}$)	1
F-13.5-1	22.5 (19.2% ${\upsigma }_{{\text{c}}}$)	13.5 (11.5% ${\upsigma }_{{\text{c}}}$)	1
F-4.5-10	13.5 (11.5% ${\upsigma }_{{\text{c}}}$)	4.5 (3.8% ${\upsigma }_{{\text{c}}}$)	10
F-9.0-10	18.0 (15.3% ${\upsigma }_{{\text{c}}}$)	9.0 (7.6% ${\upsigma }_{{\text{c}}}$)	10
F-13.5-10	22.5 (19.2% ${\upsigma }_{{\text{c}}}$)	13.5 (11.5% ${\upsigma }_{{\text{c}}}$)	10
F-4.5-20	13.5 (11.5% ${\upsigma }_{{\text{c}}}$)	4.5 (3.8% ${\upsigma }_{{\text{c}}}$)	20
F-9.0-20	18.0 (15.3% ${\upsigma }_{{\text{c}}}$)	9.0 (7.6% ${\upsigma }_{{\text{c}}}$)	20
F-13.5-20	22.5 (19.2% ${\upsigma }_{{\text{c}}}$)	13.5 (11.5% ${\upsigma }_{{\text{c}}}$)	20

## Numerical modeling

**Figure 5:**
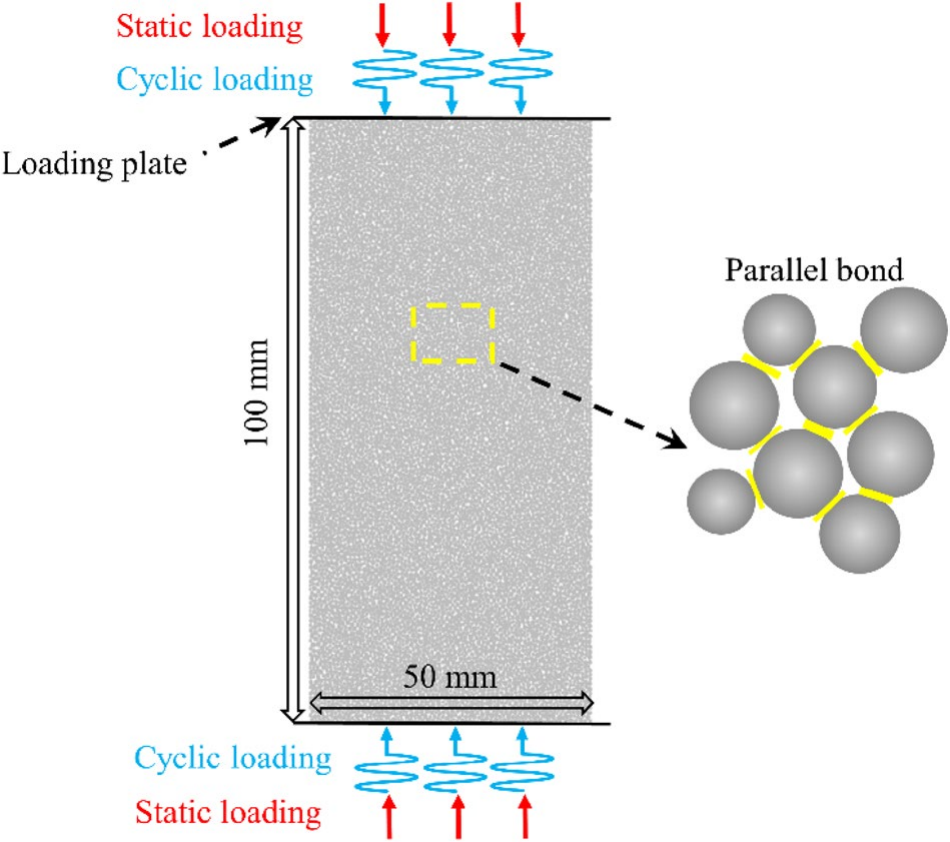
Establishment of the PFC^2D^ model.

Based on the DEM, a PFC^2D^ procedure was used to establish the numerical model of limestone and carry out the numerical simulation test under uniaxial multi-level cyclic loading. All the modeling, simulation, and analysis process were implemented in PFC^2D^. In PFC, Parallel Bonding Model (PBM) can well represent the shear, and tensile failure between particles and has significant advantages in the study of rock fracture mechanism. Therefore, PBM between particles is applied to construct the numerical specimen, as shown in Fig. 5. The model for limestone is a rectangle with a size of 50 × 100 mm. The number of particles was 12,675, and the particle radius follows a normal distribution between 0.25 and 0.42 mm.

The micro parameters of these particles mainly determine the mechanical properties of the model. Thus, the selection of microparameters greatly influences the simulation results. It is necessary to calibrate a set of proper micro-parameters in PFC^2D^ that coincide with the macro mechanical behavior. In this process, the “tried and error” method was adopted. This method refers to inputting a series of microscopic parameters into the numerical model and comparing the simulation results with the experimental results until the error between the simulation results and the experimental results is within an acceptable range. The final determined micro-parameters are listed in Table 3.

**Table 3:** Micro parameters of the numerical model.

Parameters	Value	Parameters	Value
Particle modulus/GPa	57.69	Parallel bond modulus/GPa	57.69
Stiffness ratio of particles	3.96	Stiffness ratio of parallel bond model	3.96
Friction coefficient of particles	0.5	Tensile strength/MPa	56.5
Particle density/kg m^−3^	2710	Cohesion/MPa	60.5
Radius ratio	1.67	Friction angle/°	37

**Figure 6:**
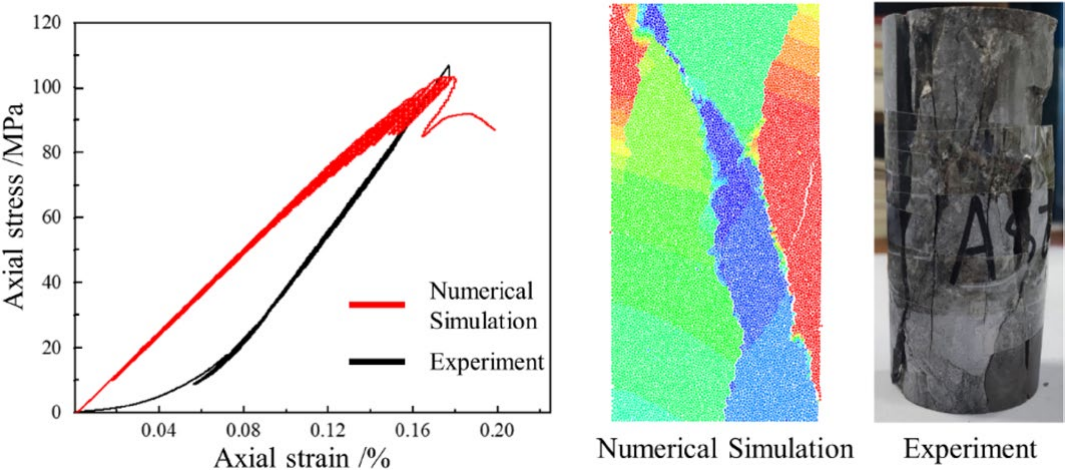
Simulation and Experiment results under uniaxial loading.

Figure 6 compares stress–strain curves and fracture mode of specimens in experiment and simulation. Different colors of particles represent fractured blocks in the specimen. It can find that the essential characteristics of stress–strain curves between experiment and simulation are consistent, except that the simulation curve lacks the initial compact deformation stage. This is caused by the compaction process of the actual rock at the initial loading stage. The fracture mode in the numerical model was similar to the experimental one. The above comparison suggests that the micro parameters can reflect the mechanical response of the limestone specimens under multi-level cyclic compression.

## Results

### Stress–strain curves

**Figure 7:**
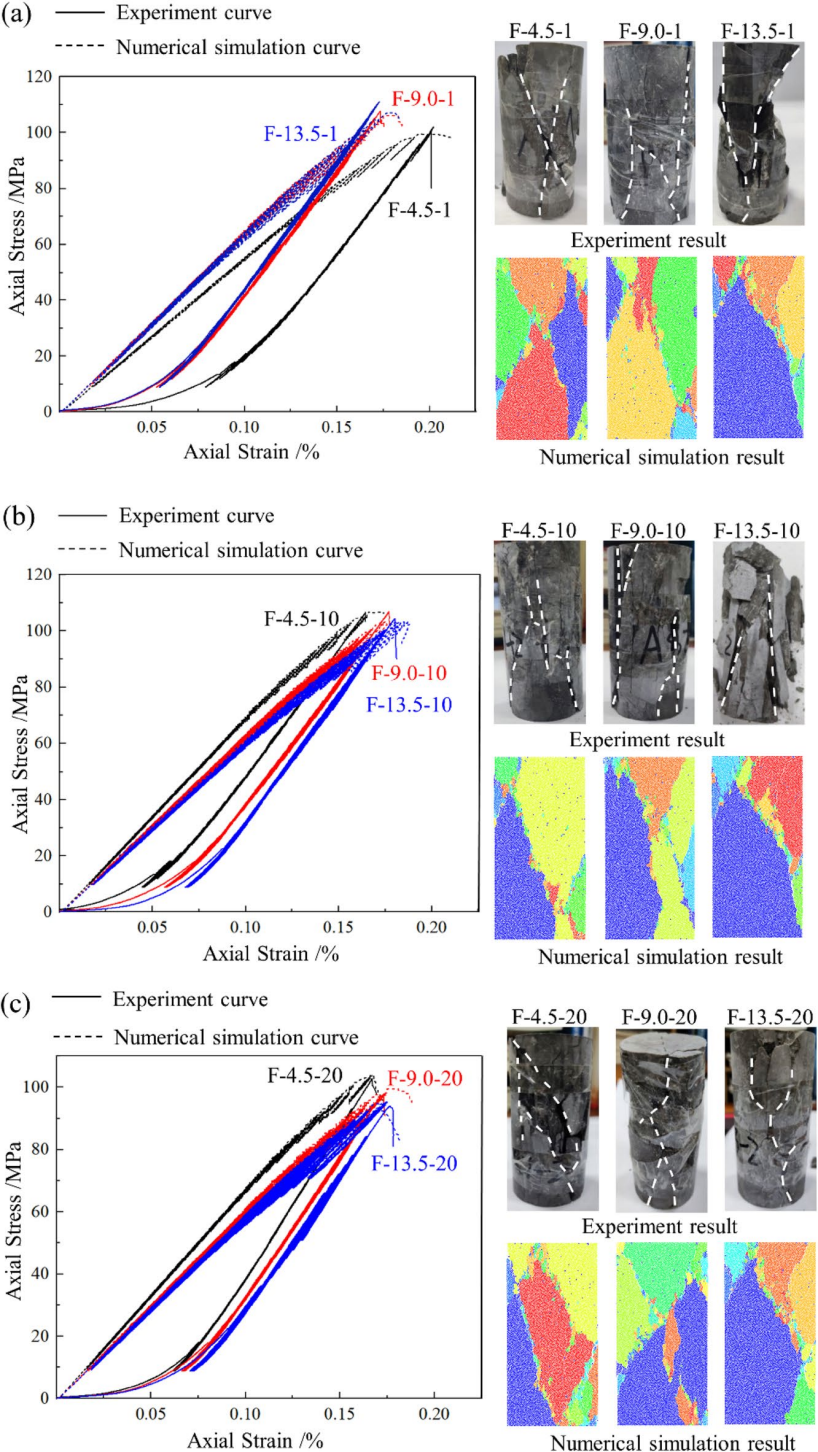
Experiment and simulation results of multi-level cyclic loading test. (**a**) *N* = 1; (**b**) *N* = 10; (**c**) *N* = 20.

Figure 7 presents the comparison results of the experimental and numerical simulation results. The curves of different colors correspond to each group's experimental and simulation results. The failure pattern of different experimental groups and corresponding numerical models are also shown in Fig. 7. The stress–strain curves obtained from PFC^2D^ show good agreement with the experimental results, except for the compaction process during loading. And the simulated failure modes of specimens were consistent with that of the experiment. Due to the fatigue effect, the area of the stress hysteresis loop increases as the cyclic loading level increases. The shape of the stress–strain curve obtained by experiment and simulation varies significantly in the final cyclic loading levels, especially when *N* = 10 and *N* = 20, indicating that plastic deformation occurs in the specimens.

**Figure 8:**
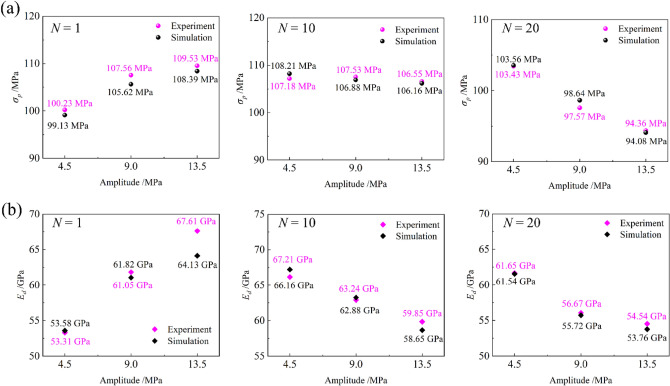
Comparison between experiment and simulation results. (**a**) Peak strength; (**b**) Dynamic elastic modulus.

Figure 8 compares peak strength (*σ*_*p*_) and dynamic elastic modulus (*E*_*d*_) under multi-level cyclic compression. As shown in Fig. 7a, *σ*_*p*_ and amplitude were positively correlated when *N* = 1. During the multi-level cyclic loading process, some of the rock fragments filled in the pores and made some of the sprouting micro-cracks closers, furtherly increasing the friction between the cracks and improving the strength of the rock. When *N* = 10 and *N* = 20, *σ*_*p*_, and amplitude were negatively correlated, the degradation effect of high amplitude on the rock became apparent. It weakened the strengthening effect caused by friction force, indicating that the long-term disturbance stress will cause more severe fatigue damage and weaken the rock. In addition, when *N* = 10 and *N* = 20, *E*_*d*_ decreased as the increased of amplitude, indicating that rock stiffness degradation under long-term multi-level cyclic loading, as shown in Fig. 7b. Therefore, the fatigue life of the rock can be prolonged only when the number of cycles in each stage changes within a specific range, which is consistent with some scholars' research results^42,43^.

### Displacement field evolution

**Figure 9:**
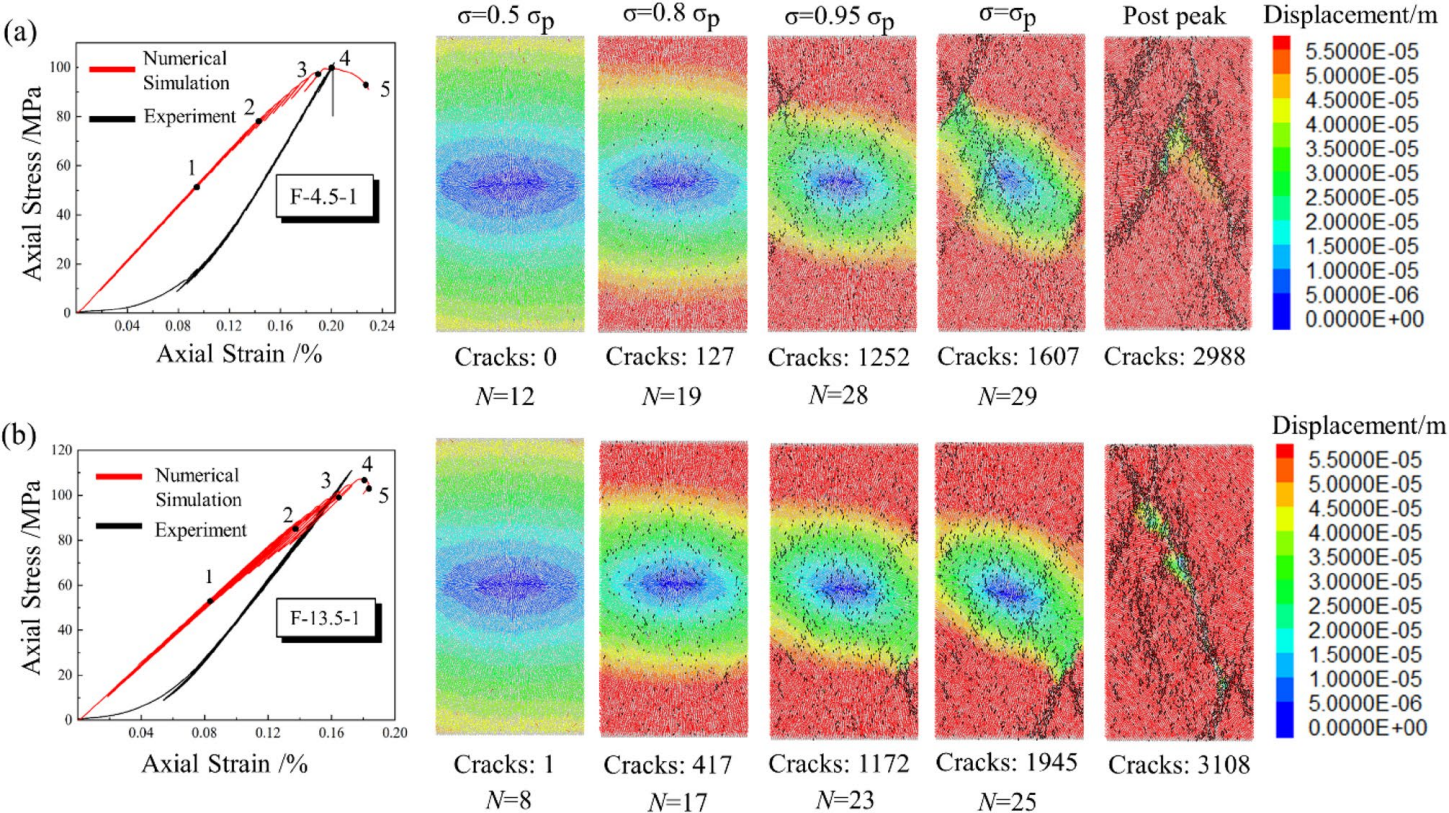
The evolution of displacement field (*N* = 1): (**a**) *A* = 4.5 MPa; (**b**) *A* = 13.5 MPa.

**Figure 10:**
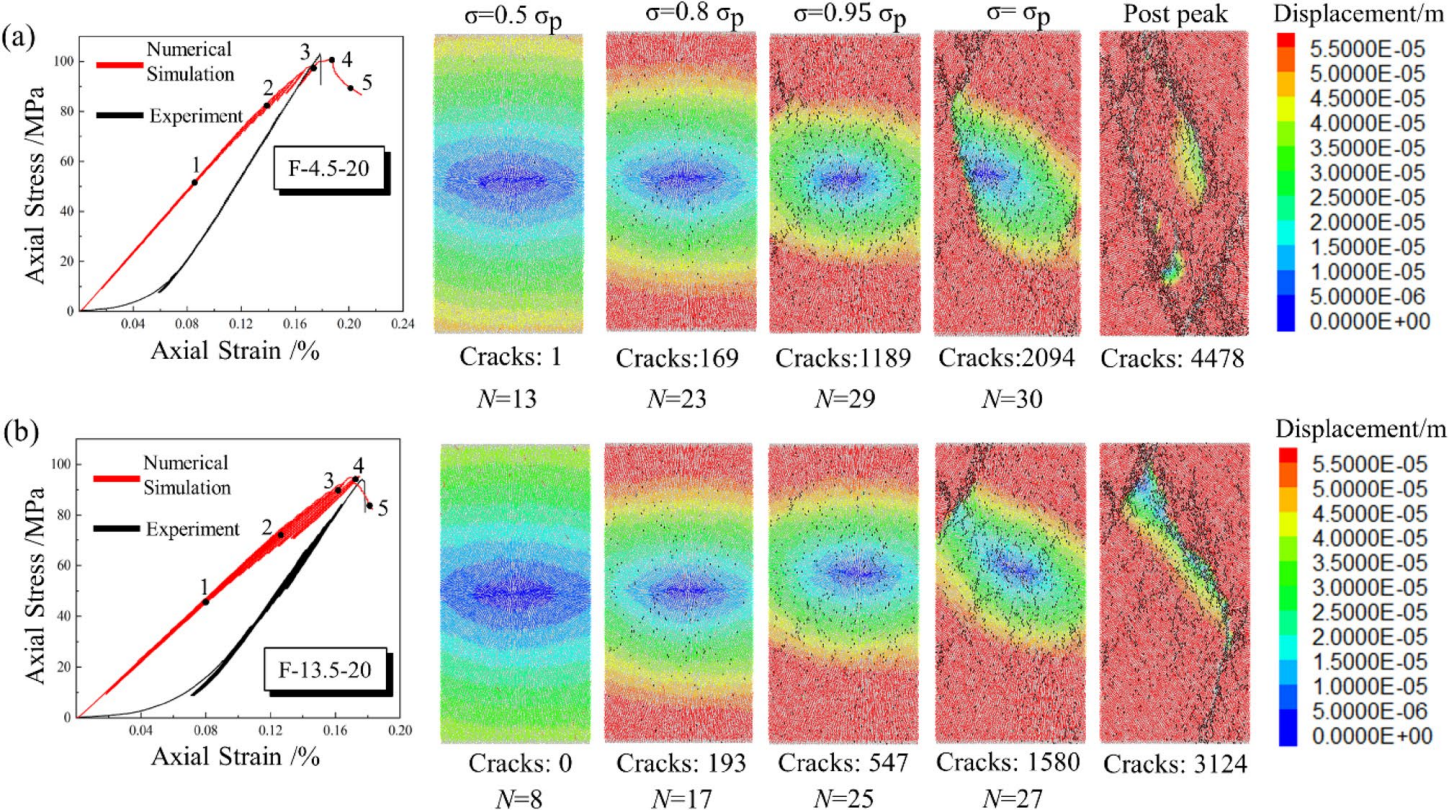
The evolution of displacement field (*N* = 20): (**a**) *A* = 4.5 MPa; (**b**) *A* = 13.5 MPa.

The crack evolution of rock is an essential mechanical response, and much research has been carried out through numerical simulation^44–46^. The displacement field method can well show the crack evolution process of the specimens during loading, as shown in Figs. 9 and 10. The black lines represent the sprouting microcracks inside the specimen. In the initial stage, the evolution pattern of each displacement field is generally consistent, and the stress–strain curve presents a linear elastic characteristic in general. Point 1 can be regarded as the dividing point between the elastic and plastic phases, and the process from point 1 to point 4 indicates the specimen’s progressive destruction. As the cyclic loading level increase, the displacement of particles at the top and bottom of the specimen gradually increase compared to those in the central area. Cyclic loading with high amplitude will cause the initial cracks to develop at a smaller stress level. When *σ* = 0.8 *σ*_*p*_, the displacement field presents a significant stratification and symmetry phenomenon, and the microcracks sprouting inside the specimen can be observed in this phase. The displacement difference between the edge and the central area is further improved as the stress level increase. Without lateral constraints, particles at the edges occur a more significant displacement than particles in the middle. Also, it can be presented that the cracks at the edges tend to propagate to the central region.

As the stress level gradually reaches *σ*_*p*_, the crack number increases dramatically, suggesting the accelerated failure stage. The propagation of microcracks affects the distribution of the displacement field in the vicinity, and cracks coalesce mainly at the position with a significant displacement difference. From point 3 to point 4, as the microcracks merge, the displacement field is split by the cracks and develops significantly. With the increase of *N*, the area of crack coalescence is also increasing. Point 5 represents the post-peak stage, driven by post-peak stress, the fracture surface slide and generate frictional resistance. The displacement field in the fractured region shrinks towards the fracture surface simultaneously, and the number of microcracks increases rapidly.

To sum up, the initial damage in the specimen happens earlier with high amplitude cyclic loading, and more microcracks will initiate in the specimen under long-term disturbance. The crack coalescence is mainly concentrated at the position where the displacement difference is significant.

### Displacement vector field

From the microscopic aspect, rock damage happens when the external stress exceeds the fracture strength between the particles. PFC^2D^ records the ultimate direction and magnitude of the particle displacements in the crack zone during simulation, such as the displacement vector field, which can present the ultimate failure modes.

**Figure 11:**
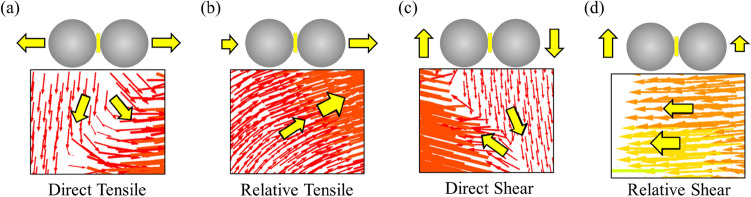
Four displacement modes between particles in PFC^2D^.

Based on the previous research^47,48^, the particles’ displacement vector can be divided into four modes according to the direction and magnitude, as shown in Fig. 11. From Fig. 11a, two groups of particles move in the opposite direction that is perpendicular to the contact surface and form direct tensile cracks; From Fig. 11b, two groups of particles move in the same direction that is perpendicular to the contact surface, but their displacements are significantly different and form relative tensile cracks; From Fig. 11c, two groups of particles move in the opposite direction that is perpendicular to the contact surface and forms direct shear cracks; From Fig. 11d, two groups of particles move in the same direction that is perpendicular to the contact surface, but their displacements are significantly different and form relative shear cracks.

**Figure 12:**
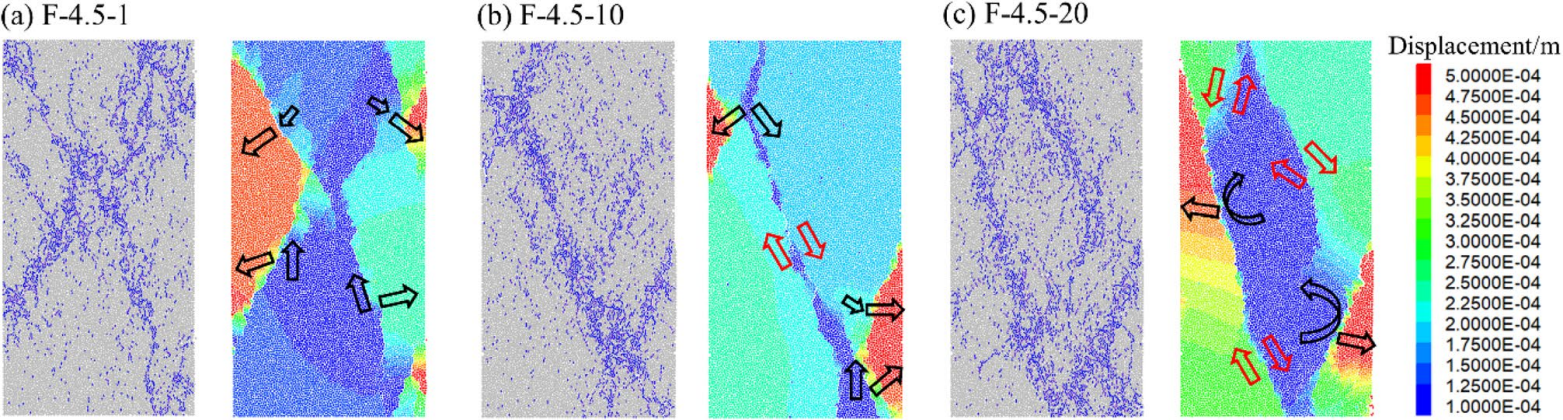
Crack distribution and displacement vector field of rock specimens (*A* = 4.5 MPa).

**Figure 13:**
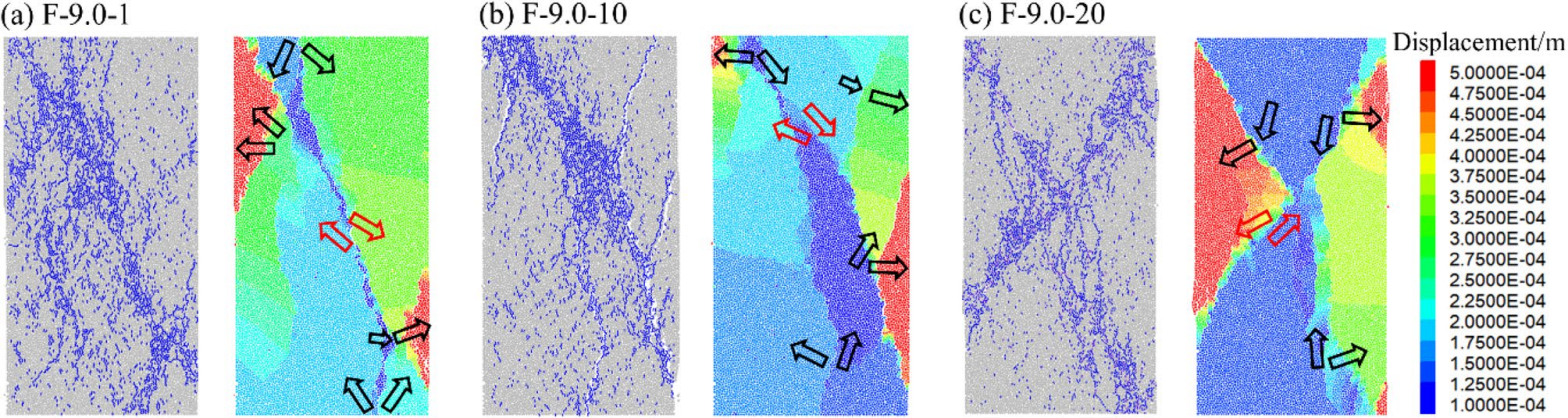
Crack distribution and displacement vector field of rock specimens (*A* = 9.0 MPa).

**Figure 14:**
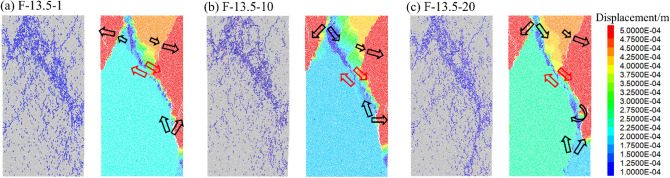
Crack distribution and displacement vector field of rock specimens (*A* = 13.5 MPa).

Figures 12, 13 and 14 displays the displacement vector field of each numerical model after failure, and the corresponding crack distribution pattern are presented. Different colors represent the value of displacement during loading. Red arrows and black arrows indicate shear cracks and tensile cracks, respectively.

In Fig. 12, when *A* = 4.5 MPa, the cycle number increase significantly influenced the cracks’ distribution pattern. Driven by direct tensile and relative tensile, particles at the edge occur significantly displacement compared to those in the central area when *N* = 1. When *N* = 10, the failure mode of the specimen is under the impact of direct shear and tensile cracks. The tensile cracks are mainly distributed at the edge of the specimen. When* N* = 20, multiple shear cracks are distributed inside the specimen, consistent with the failure modes in Fig. 7.

Figure 13 shows the displacement field when *A* = 9.0 MPa. As the amplitude increase, the failure mode with mixed tensile-shear cracks is generally presented. When *N* = 1, the failure mode is similar to that in Fig. 12b. However, with the increase of N, the specimens displayed a complex failure pattern consistent with the crack distribution in Fig. 7. Dominated by multiple tensile displacement fields, the specimens produce multiple tensile cracks extending from the edges to the central area. The zigzag cracks improve the resistance of the specimen to deformation. In contrast, the crack intersection region is mainly affected by the direct shear displacement field, where so many micro cracks are distributed, implying that when the penetration of macroscopic cracks in the rock occurs, it is accompanied by the misalignment effect between multiple rock masses.

Figure 14 presents that when A = 13.5 MPa, Y-shaped cracks appeared. The increase of *N* had little effect on the failure mode of the rock. The cracks at the specimen edge are mainly induced by the direct tensile and relative tensile cracks. The shear displacement fields are primarily distributed in the confluence region of the tensile cracks and result in a significant slip surface when the specimen fractures, which is consistent with the failure mode shown in Fig. 7. It can be considered that shear cracks are generated by coalescing microcracks distributed at the edge of limestone.

It is worth noting that some particles show a rotation track in the displacement process, which is caused by the loosening of particles in this area during the fracture process, usually accompanied by tensile failure. In summary, specimens exhibit predominantly tensile cracks when *N* and *A* are low under multi-level cyclic loading conditions. As *N* increases from 1 to 20, the failure mode of the rock tends to be more complex. Cracks generated by the shear cracks mainly cause dislocations between rock masses. The primary failure mode of the specimens at a lower *A* presents a transformation from tensile failure to mixed failure, and the failure modes change little when *A* = 13.5 MPa.

## Conclusions

A series of multi-level cyclic loading experiments were performed on limestone specimens. Based on the experiment results, the corresponding numerical simulation was implemented in PFC^2D^. This study was carried out from the perspective of stress–strain curves, the evolution of the displacement field, and the vector field of displacement. Some main conclusions are drawn as follows:

1. The strengthening and deterioration of limestone are affected by the number of loading cycles. When the number of cycles is low, the increase in the amplitude has a strengthening effect on the limestone. As the cycle number increases, the strength and stiffness of limestone will exhibit more obvious deterioration characteristics with the amplitude increase.
2. From initiation to coalescence, the process of crack evolution in the specimens is thoroughly captured by PFC^2D^. The stress level when crack initiation decreases with amplitude increasing, while the improvement of cycle number induces more irreversible damage. The crack coalescence is caused by the increase in the displacement difference between the edge and the central area, and cracks propagate from the edge of the model to the central region.
3. Typical failure mode of the limestone was identified. Limestone mainly suffers tensile failure when the cyclic disturbance is not significant. With the improvement of loading cycles, failure mode with mixed cracks will become universal. Limestone generally exhibits a failure mode of shear crack penetration under high-amplitude disturbance, where tensile cracks are mainly distributed at the edge of the specimens. Shear cracks are formed by the penetration of tensile cracks distributed on the edge of the rock and cause the slippage and dislocation of the rock blocks.

## Data Availability

All data included in this study are available upon request by contact with the corresponding author.
